# A Medical Naval Volunteer on Duty at Quebec, July, 1908

**Published:** 1908-12

**Authors:** W. Kenneth Wills

**Affiliations:** M.B., B.C. Cantab.


### A MEDICAL NAVAL VOLUNTEER ON DUTY AT QUEBEC, JULY, 1908.

BY

W. KENNETH WILLS, M.B., B.C. Cantab.

---

THE ways of the Admiralty are inscrutable, but though it may be the general service opinion that the main function of that august body consists in being adversely criticised, I found no inclination to follow suit when I discovered that I was gazetted to the *Russell*, and that my destination was Quebec.

It was pardonable that I was both in high spirits and a quandary at one and the same moment. For I had been refused the trip to Quebec already by the Admiralty Volunteer Committee on the plea of no room, and I had taken their decision that I should proceed forthwith to Portsmouth to join the *Vengeance* for manœuvres as " one of the laws of the Medes and Persians that altereth not." In the *Vengeance*, however, a signal found me which sent me off express to Devonport to join the *Russell*; but it only dawned slowly upon my awakening intelligence department that the *Russell* and I were bound for Quebec. This meant six weeks from home, when I had only arranged for four : hence the quandary !

On arriving at Devonport, I discovered that the *Russell* had already started for Berehaven. A night or two in barracks was the result of this, which gave one an opportunity of looking in at the hospital quarters there, and I only just managed to avoid being inspected by my Lords of the Admiralty, who were making a personally-conducted tour of the premises.

I obtained a passage in the *Duncan* to Berehaven, and in the ship met five other medicals, some belonging to the *Duncan*, others bound, like myself, for the other ships of the Atlantic Fleet. One of these was Surgeon Cock, R.N., of the *Russell*, who, in the way they have in the Navy, immediately took me in tow, and commenced the hospitality which Staff-Surgeon Philip and he maintained throughout the cruise in the most cordial fashion. The N.O. (naval officer) has reduced the science of hospitality to a fine art, and if one is not at home in a ship within a short time of joining, there is something wrong with the " cut of one's own jib." What greater test could there have been of hospitality than was exemplified by one of the officers lending me his golf clubs before even I had been introduced to him ? so that I was able to contest an even match with Cock over Bere Island golf links (save the mark !).

We put to sea on July 5th, on an even keel and in brilliant weather, and at " economic " speed soon left the " Bull " and " Calf " behind us, and entrusted ourselves to the " Baths of Ocean," *en route* for the Hesperides.

We comprised in " line ahead " the first-class battleships *Exmouth* (flagship), *Albemarle, Duncan, Russell,* and the cruisers *Arrogant* and *Venus.*

The passage out was uneventful. There was little to do in the sick bay, and the problem was how to keep clear of the ship's routine, and yet do anything worth doing. It soon began to grow foggy and cold, and we were all on the look-out for icebergs. There was nothing to do on deck, and nothing to do below. It was bitterly cold on deck, where everything dripped and the quarter-deck awning leaked ; and it was stifling in one's cabin, which being on the waterline could not " carry the scuttle." The wardroom and the smoking casemate were the sole refuges of the destitute N.O., and even these were not always reliable. At gun-drill the casemate was dismantled, and at turret (12-in.) drill all the dead-lights of the wardroom skylights were down, leaving midnight below. Noise, eruptions, interruptions, sirens with Cheyne-Stokes rhythm or suffering from sporadic attacks of Morse-code cough, " general quarters," " fire stations," " collision stations," " desert ship," or " night quarters "—these were strenuous times for those concerned, but how any reading can be done whilst afloat is not easy to imagine. Yet it *is* done, and the surgeon manages to circumvent these difficulties, and strenuously attempts to keep up to date. All honour to him who succeeds !

As we neared the Straits of Belle Isle we met icebergs, and our first sight of one of these was in brilliant sunshine on an ultramarine sea. It was only a small one, but it was peculiar in that it was a silhouette of Mr. Chaplin, so exactly reproduced that it might have been a marble effigy of that parliamentarian.

We had our first medical case of any importance at this time, one of the stokers arriving in the sick bay with an indefinite form of pneumonia. There were few physical signs, and the general symptoms out of all proportion to the extent of the pulmonary trouble. He started with an attack of unconsciousness, with low muttering delirium, but gradually improved, very slight evidences of rusty sputum occurring some days later. As another case very similar in nature occurred while we were at Quebec among the wardroom servants, it seems worthy of notice. Both did well.

The weather improved as we drew nearer our destination, and we bade farewell to the snow lying on the heights of Labrador, and thanked Heaven that it was 90° in the shade at Quebec.

On the 14th we reached the Traverses, south of the Island of Orleans, so famous in the story of the progress of Saunder's fleet up the unknown St. Lawrence, and passing round the Levis shore, we opened out the panorama of Quebec, just as the sun was setting behind the heights of Abraham.

There can be few more striking pictures than met our eyes at that moment. Quebec was framed by the setting sun, and the evening haze made the distance more effective and mystical, till citadel and fortress, church and seminary, hotel and dwelling-house, warehouse and even elevator became the battlements of some enchanted, castellated and impregnable city. Behind towered the heights of Abraham ; to the north the blue uplands of the Laurentian Shield ; far away to the right the white lace falls of the Montmorency slipping into their silent depths ; to the left the Levis shore, to which nearness gave contrasting detail, while the broad sweep of the St. Lawrence, widened out as it is into an inland sea by the junction with it of the Charles River, reflected the whole as in a mirror of gold. The effect is not likely to be effaced from one's memory, nor could one help wondering if such was the sensation that Wolfe experienced as his transports passed into the view of that impregnable fortress, and whether even his intrepid heart sank at the stupendous task before him.

Our flotilla formed into "double line ahead," and quietly dropped anchor in midstream beneath the citadel. Next morning the powder began to burn in salutes to the port, salutes from the port, and salutes to officials exchanging visits. But this was only the preliminary canter, for the French ships had not arrived, nor yet the American, nor the *Indomitable* and *Minotaur*.

By the courtesy of the staff-surgeon, I obtained shore-leave, and investigated Quebec and the Montmorency Falls. Quebec itself was transformed into an old-time city with the costumes of centuries ago, for by special request the mummers of the pageant wore their distinctive dresses about the streets. The illuminations belonged, however, to the twentieth century, for the main buildings were tricked out with electric lights in profusion, giving a delightful effect when viewed from the river.

Five days yet remained before H.R.H. the Prince of Wales was timed to arrive, so the surgeon of the *Russell* and I, obtaining shore-leave for four days, " did " Niagara, Toronto and Montreal.

On our return from a most enjoyable though far too " hustled " a trip, we found that the American and French warships had arrived, and also a Canadian river gunboat. The *Russell* was to entertain the officers of the *Amiral Aube* on the following day, so there was a kind of census taken of those officers who could talk French. The result was disheartening: one interpreter and a midshipman or two comprised our Franco-linguistic strength, though not a few seemed strong in language in general! But when our guests arrived and were found to understand not a word of English, desperate attempts were made which were not wholly unsuccessful. (By the by, the French idea of a teetotaller is one who drinks tea with every meal!)

One of our guests was the ship's surgeon, who was insatiably interested in the sick quarters and the service-afloat stores. His eyes gloated over the bacteriological and water-testing case, but he preferred the French arrangement of a double sick bay—on the port side for out-patients, and on the starboard for cots, operations, &c. With us, of course, there is one large sick bay, which can be divided off by screens as required. One surgeon is deemed sufficient apparently for a French man-of-war. To carry three medicals was thought to be a great excess, and when it was explained that one was a " volontaire," our medical guest thought (as I did) that it was a paternal Government that allowed such a thing.

On July 22nd the *Indomitable* and *Minotaur* arrived with the Prince, and every gun in the ships and citadel spoke the welcome with no uncertain voice.

Quebec was crowded by this time, and necessaries had reached famine prices, equalling or surpassing the days of Bigot the Intendent and La Friponne!

The militia battalions of Canada were encamped in thousands in the neighbourhood, there was a huge tented city for visitors, and there was not a bed to be had in the neighbourhood. So it cost a dollar to be shaved, and one N.O. who lost his last boat off to his ship was accommodated with a shakedown on a hotel table for the small cost of fifteen dollars for the night, just to show that there was no ill-feeling between the French-Canadians and their English brethren. Another instance of their elastic ways appeared in our dealings with the laundry. The tariff supplied to the officers of the fleet attracted the linen from the ships in quantity. When it was in hand the prices suddenly rose to a ruinous figure. No redress was obtainable from sympathising magistrates. However, the marine is as subtle as the Chinee at times, and one servant, on applying for his master's washing, was handed the parcel, and a sum was demanded in payment. The marine objected to the amount as not according to agreement. "You pay that or nothing," came the response. "Now we know where we are," said the marine, and left hurriedly. Thus did Lamb's anecdote of the Quakers and the breakfast-lunch bear fruit in after years.

The Prince's reception, however, left no doubt in the mind that the French-Canadians were British subjects, with a capacity for enthusiasm truly British in quality. The East joined hands with the West in a remarkable demonstration of greeting, and from this time on there was no cessation of functions. The pageant was, perhaps, the chief attraction of the Tercentenary, but the Grand Review was the most noteworthy. In this some 20,000 or more naval and military troops took part on the Plains of Abraham, including the British, American and French bluejackets, the marines, and the Canadian militia. By special request of the Prince the Naval Volunteers were included in the review, and made a very good account of themselves.

The personnel surrounding the Prince on this occasion was of great interest. The Duke of Norfolk, Lord Roberts (who obtained an ovation nearly equal to that of the Prince himself), the Governor-General of Canada, Vice-President Fairbanks (U.S.A.), Admiral Jaureguiberry and other French representatives, the Earl of Dudley (on his way to Australia to assume the office of Governor-General), Sir Wilfred Laurier, and the topographically interesting lineal representatives of those who made Quebec famous: Mr. George Wolfe, the Marquis de Levis, the Marquis Levis-Mirepoix, the Comte de Montcalm, Capt. the Hon. Arthur

Murray, the Hon. Dudley Carleton, and the Mayor of Brouages, the birthplace of Champlain.

The occasion of the review was made the opportunity for the Prince to hand over to the nation the Quebec battlefields as a national park, with the money collected for the purchase and improvement.

On " Champlain Day " there appeared in the river, under full sail, the little *Don de Dieu*, not much larger than a masted picket-boat, with a high poop, and looking microscopic by the side of the *Indomitable.* This was a facsimile of that ship which, 300 years ago, brought Champlain to Quebec. Now, as then, Red Indians in feathers and war-paint paddle out their canoes to investigate this strange and sinister apparition. Being satisfied, however, on this occasion that he is coming peaceably, they escort Champlain to the shore, where he proceeds to his " habitation " erected in the town. As near a representation as possible was made of this, though the stockade surrounding it was, for the purpose of observation, a great deal lower than the original must have been.

The day, celebrated with many speeches and much congratulation, was concluded by a grand display of fireworks from the Levis shore, which after a hopeful beginning came to an untimely end in about twenty minutes, to the great disappointment of the assembled thousands. About half an hour later, however (when the French officers had really finished their dinner), the pyrotechnic display recommenced, and from the noise and length of time expended over it must have been a very successful show. Unfortunately, the wind set in our direction, so that we obtained a view of the smoke and little else.

The pageant on the Plains of Abraham was an unqualified success. No expense or trouble had been spared, and when it is considered that some 4,000 persons took part in producing the various scenes it will be conceded that the Master of the Pageant (Lascelles) had no light task before him when he undertook the post of Director of Ceremonies. The brilliant success of each scene amply repaid the trouble and care expended upon it, for colour and scenery, costume and effect were blended in a manner both picturesque in themselves and highly suitable to the beautiful position chosen for the pageant.

Thus was it possible to lose oneself in retrospect upon historic ground, and with a mind's eye subject to anachronismal astigmatism to fuse together in a few short hours the story of Quebec, from the landing of Champlain in 1608 to the time of Wolfe and Montcalm, while the spirit of the people present told of fresh history in the making—French and British together, in remembrance of a brave past, uniting in one common hope and interest for the future under the flag of the Dominion.

The visit to Quebec was not devoid of its elements of humour. Did not the amorous N.O. catch the answering glance of the costumed beauty, and expend most of the evening in ascertaining that the seventeenth-century costume does as well for a boy as for a girl! Did not two N.O.'s journey out to the militia encampment and lunch with the Highlanders, and respond to their many and full-throated cheers for the British Navy with the best they could do! And did not the whole naval flotilla succumb to a night torpedo attack, which the searchlights wandering in every direction "failed" to discover! Did not all the ships in the river blow up simultaneously in realistic fashion with a hundred rockets apiece from the several forecastles, at the expense of the British taxpayer, while the guns made as much noise as possible, according to Admiral's order, "because the ladies like it!"

Balls, receptions, garden-parties and ship-parties made the time pass all too soon, and hearts were lost at Quebec that took at least two days and the prospect of the Dublin Horse Show to heal again.

One incident may be added which reveals the other side of the brave N.O. The cruiser *Venus* had been detailed on the voyage up the river to help drag a collier off the rocks which had piled herself up in a fog. On taking up her station later on the cruiser crossed the bows of the *Russell* on change of tide. Somehow the eight-knot tide drove her on to our bows while we were at dinner. When a noise of a heavy door banging forward was heard and the ship trembled, we thought that the marine party responsible for closing water-tight doors in the evening were getting tired, and had let one of these hatches down with a run. But when the *Venus* shaved our port-side at an eight-knot speed we saw that an accident had occurred. Orders were promptly given and carried out as to water-tight doors, &c., and then it was ascertained that the after-casemate 6-in. gun in the *Venus* had fouled the lip of our hawse-pipe, hurling itself thirty feet back into the ship and breaking off her trunnions. Had not the *Venus* rotated herself off our bows in this providential manner she and our ram must have become acquainted. The importance, of course, lay in the "might have been," but the quiet contained demeanour of the officers in charge tells of the hero lurking in every man who only appears "as occasion requires."

We escorted the Prince to the mouth of the St. Lawrence, and gave him a send-off on his record passage home. For ourselves, we threaded our economic path homewards through icebergs and fog till we reached Erin's Isle in sunshine with regret that the cruise was over, and the last we heard of the hospitable *Russell* was the deep-voiced song of the 12-in. guns "calibrating" off Berehaven.

